# Advances in Interventional Oncology for Hepatocellular Carcinoma

**DOI:** 10.3390/cancers18203319

**Published:** 2026-10-14

**Authors:** Sameer Gadani, Edwin Chou, Jirapa Chansangrat, Hashim Salar, Baljendra Kapoor

**Affiliations:** 1 Interventional Radiology Division, Imaging Institute, Cleveland Clinic Foundation, Cleveland, OH 44195, USA; gadanis@ccf.org (S.G.); edwinchou@gmail.com (E.C.); 2 School of Radiology, Institute of Medicine, Suranaree University of Technology, Nakhon Ratchasima 30000, Thailand; jirapacha1986@gmail.com; 3 Medical College, Faculty of Health Sciences, Aga Khan University, Karachi 74800, Pakistan; hashim.salar@scholar.aku.edu; 4 Department of Radiology, University of Michigan, Ann Arbor, MI 48109, USA

**Keywords:** hepatocellular carcinoma, interventional oncology, locoregional therapy, transarterial radioembolization, transarterial chemoembolization, percutaneous ablation, immunotherapy, combination therapy

## Abstract

Locoregional therapies are used across the hepatocellular carcinoma (HCC) treatment spectrum, including for curative treatment of small tumors, as bridging or downstaging therapy before liver transplant, for treatment of intermediate-stage disease, and as part of combination treatment with systemic therapy in selected patients. This narrative review summarizes recent developments in transarterial radioembolization (TARE), transarterial chemoembolization (TACE), and percutaneous ablation techniques and separates established clinical advances from early investigational work. The strongest evidence supports personalized TARE dosimetry and radiation segmentectomy, selective TACE with reassessment before repeat treatment, and image-guided thermal ablation with objective margin assessment. Phase 3 trials of TACE combined with antiangiogenic and immune checkpoint therapy demonstrated improved progression-free survival in selected patients with unresectable, nonmetastatic HCC, although the survival benefit of these combinations so far extends only to progression-free survival, and the best treatment sequence remains to be defined. Histotripsy, pulsed electric-field ablation, electrochemotherapy, cryoablation, high-intensity focused ultrasound, imageable or alternative radioembolic microspheres, new embolic agents, robotics, and artificial intelligence remain at different stages of clinical development. Most supporting data are derived from single-arm studies, retrospective cohort studies, trials in mixed liver-tumor populations, or studies in preclinical models. Treatment selection should account for tumor burden and location, liver function, portal hypertension, vascular invasion, transplant candidacy, available alternatives, local expertise, and patient preferences. Future studies should focus on durable local control, preservation of liver function, quality of life, transplant eligibility, overall survival, and cost.

## Simple Summary

Liver cancer is one of the most common causes of cancer death worldwide, and most cases begin in a liver already damaged by long-standing disease. This makes treatment difficult, because destroying a tumor must not tip a fragile liver into failure. Over the past decade, doctors have refined image-guided treatments that target liver tumors directly delivering tiny radioactive beads, blocking a tumor’s blood supply, or destroying it with heat, cold, or sound waves often without surgery. This review gathers recent progress in these therapies and, importantly, separates approaches now supported by strong evidence from those that remain experimental. It also examines how these local treatments can be paired with newer whole-body drugs. Our aim is to help clinicians choose treatments that control cancer while protecting liver function and quality of life, and to highlight where better studies are still needed.

## 1. Introduction

Primary liver cancer remains a major cause of cancer mortality worldwide, and hepatocellular carcinoma (HCC) accounts for most primary liver cancers. Because HCC usually develops in a chronically injured liver, treatment must balance tumor control against the risk of hepatic decompensation. Current staging systems and practice guidelines therefore take into account tumor burden, liver function, performance status, portal hypertension, and the feasibility of various treatment options [1,2,3]. Better outcomes over time in patients with HCC reflect earlier detection of disease, increased implementation of antiviral and cirrhosis care, improved imaging, and increased use of transplant, surgery, locoregional therapy, and systemic therapy [2,4].

Interventional oncology now has a role in early, intermediate, and selected advanced HCC cases. Ablation is a curative-intent option for appropriately selected small tumors, and recent data have shown that this technique leads to outcomes comparable to those seen with surgery in selected patients. Transarterial chemoembolization (TACE) remains a standard treatment for many patients with intermediate-stage disease, and transarterial radioembolization (TARE) can be used for radiation segmentectomy, lobar disease control, bridging therapy or downstaging, and treatment of selected patients with portal venous invasion [1,5,6,7].

This narrative review focuses on minimally invasive and noninvasive interventional treatment strategies for patients with HCC. Guidelines and randomized trials are given the greatest weight, followed by prospective comparative studies, well-conducted cohort studies, and systematic reviews. Single-arm trials, retrospective series, and preclinical studies are used mainly to describe feasibility and future directions. Note that technical success, objective response, and progression-free survival are important endpoints, but they do not substitute for the endpoints of overall survival, preserved liver function, quality of life, or successful transplant.

### Scope and Search Strategy

This is a narrative review. We searched PubMed/MEDLINE and Embase for English-language articles published from January 2002 to the date of final revision. The search combined terms for hepatocellular carcinoma with terms for interventional oncology, locoregional therapy, radioembolization or TARE, chemoembolization or TACE, ablation (radiofrequency, microwave, cryoablation, and irreversible electroporation), histotripsy, pulsed electric field, high-intensity focused ultrasound, and immunotherapy combinations. We gave most weight to guidelines and randomized trials, then to prospective comparative studies, well-conducted cohorts, and systematic reviews. Single-arm trials, retrospective series, and preclinical work informed the sections on feasibility and future directions. We also screened the reference lists of key articles. The corresponding author should confirm the databases searched and the final search date.

## 2. Patient Selection and Multidisciplinary Decision-Making

The same radiologic tumor burden can represent different clinical problems. Therefore, several factors should be considered when selecting a patient for locoregional therapy, including Child–Pugh class or albumin–bilirubin grade, presence or absence of portal hypertension, bilirubin level, presence or absence of ascites, presence or absence of encephalopathy, renal function, performance status, tumor number and distribution, proximity of tumor to bile ducts and bowel, presence or absence of macrovascular invasion, presence or absence of extrahepatic disease, and volume of uninvolved liver. Both the Child–Pugh class and the albumin–bilirubin (ALBI) grade can be used to gauge liver reserve. Child–Pugh class incorporates ascites and encephalopathy but the scoring can be partly subjective; the ALBI grade is objective and better discriminates hepatic reserve within Child–Pugh class A, so it is often preferred when stratifying candidates for locoregional therapy, particularly before radioembolization [8]. For that reason, many teams favor ALBI when selecting candidates for locoregional therapy, and particularly before radioembolization. Multidisciplinary review is most useful when more than one potentially curative option is available or when treatment outside a strict stage-based recommendation is being considered. In practice, the anatomy, treatment intent, liver reserve, and expected durability of treatment often determine the choice of intervention rather than disease stage alone [1,2].

Treatment intent should be documented before the procedure. For patients with early HCC, the intent may be curative, bridging to transplant, or downstaging to allow for transplant or resection. For those with intermediate-stage disease, the goal may be to achieve durable intrahepatic control while preserving eligibility for later systemic therapy or transplant. In cases of advanced disease, locoregional treatment should be reserved for patients in whom the expected benefit outweighs the risk of delaying effective systemic therapy [9].

Repeat locoregional treatment should not be performed automatically; rather, it should be performed based on response and liver function. Rapid intrahepatic progression, worsening liver function, new vascular invasion, extrahepatic spread, or lack of meaningful response to treatment should prompt reassessment and, when appropriate, a change in locoregional modality or transition to systemic therapy. This is particularly important after nonselective transarterial treatment, which can reduce liver reserve without providing durable benefit [1,2,9].

Interventional therapies belong inside the standard staging-based algorithms. Within the Barcelona Clinic Liver Cancer (BCLC) 2022 framework and AASLD and EASL guidance, ablation offers curative-intent treatment for very early and early disease, TACE is the standard for intermediate-stage disease, and TARE serves several roles that include radiation segmentectomy, lobar control, bridging or downstaging, and treatment of selected vascular invasion [2,10]. Systemic therapy anchors advanced disease and is used more and more alongside locoregional treatment. Table 1 shows how the modalities discussed below fit this framework. It also shows that the decision within a given stage turns on anatomy, treatment intent, liver reserve, and the durability a patient needs, so stage alone rarely settles the choice.

## 3. Advances in Locoregional Therapies

Recent clinical progress has come mainly from better treatment planning and verification rather than from new devices alone. Across TARE, TACE, and ablation, factors that must be considered include appropriate patient selection, use of selective treatment, achievement of adequate dose or margin, and early confirmation that the intended target was treated.

### 3.1. Personalized TARE Dosimetry and Radiation Segmentectomy

TARE delivers beta-emitting microspheres through the hepatic arterial supply. Earlier activity calculations often used a single-compartment model that assumed relatively uniform microsphere distribution within the perfused volume; however, HCC frequently demonstrates preferential arterial flow. Therefore, multicompartment or partition models are now often used to estimate tumor and nontumoral absorbed doses separately. The practical advance with this method is a patient-specific plan that links angiographic distribution, treatment volume, microsphere characteristics, and absorbed dose [11].

The phase 2 DOSISPHERE-01 study provided evidence supporting this personalized dosimetry [12]. In patients with locally advanced HCC, TARE interventions targeting at least 205 Gy to the index lesion produced a higher objective response rate and more downstaging than interventions using standard dosimetry, with a substantial increase in median overall survival. These findings demonstrated that absorbed tumor dose is a modifiable treatment variable. Note that this study was relatively small and used glass microspheres, so the numerical threshold should not be generalized to other devices or dosimetry methods.

The phase 3 SARAH trial, which compared sorafenib treatment with resin-microsphere TARE interventions planned using conventional methods, did not demonstrate superior overall survival [11]. However, a later dosimetric analysis demonstrated a strong association between higher tumor-absorbed dose and both response and survival [13]. This post hoc analysis did not provide randomized proof, but it highlighted why tumor absorbed dose should be reported rather than administered activity alone.

Radiation segmentectomy applies the same principle of personalized dosimetry to curative-intent treatment. With radiation segmentectomy, an ablative radiation dose is delivered to one or two hepatic segments. An ablative dose here generally means a single-compartment (perfused-volume) absorbed dose of at least 190 Gy. In current practice, glass microspheres are often taken to a considerably higher target, on the order of 400 Gy, while resin microspheres are used at roughly 190 to 200 Gy [14,15]. This technique can be useful for tumors that are unsuitable for thermal ablation because of size, location, visibility, or proximity to vessels. The LEGACY cohort study and the prospective RASER study reported high rates of durable local control and complete imaging or pathologic response in selected patients with solitary early-stage HCC who were treated with radiation segmentectomy [14,15].

**Figure 1:**
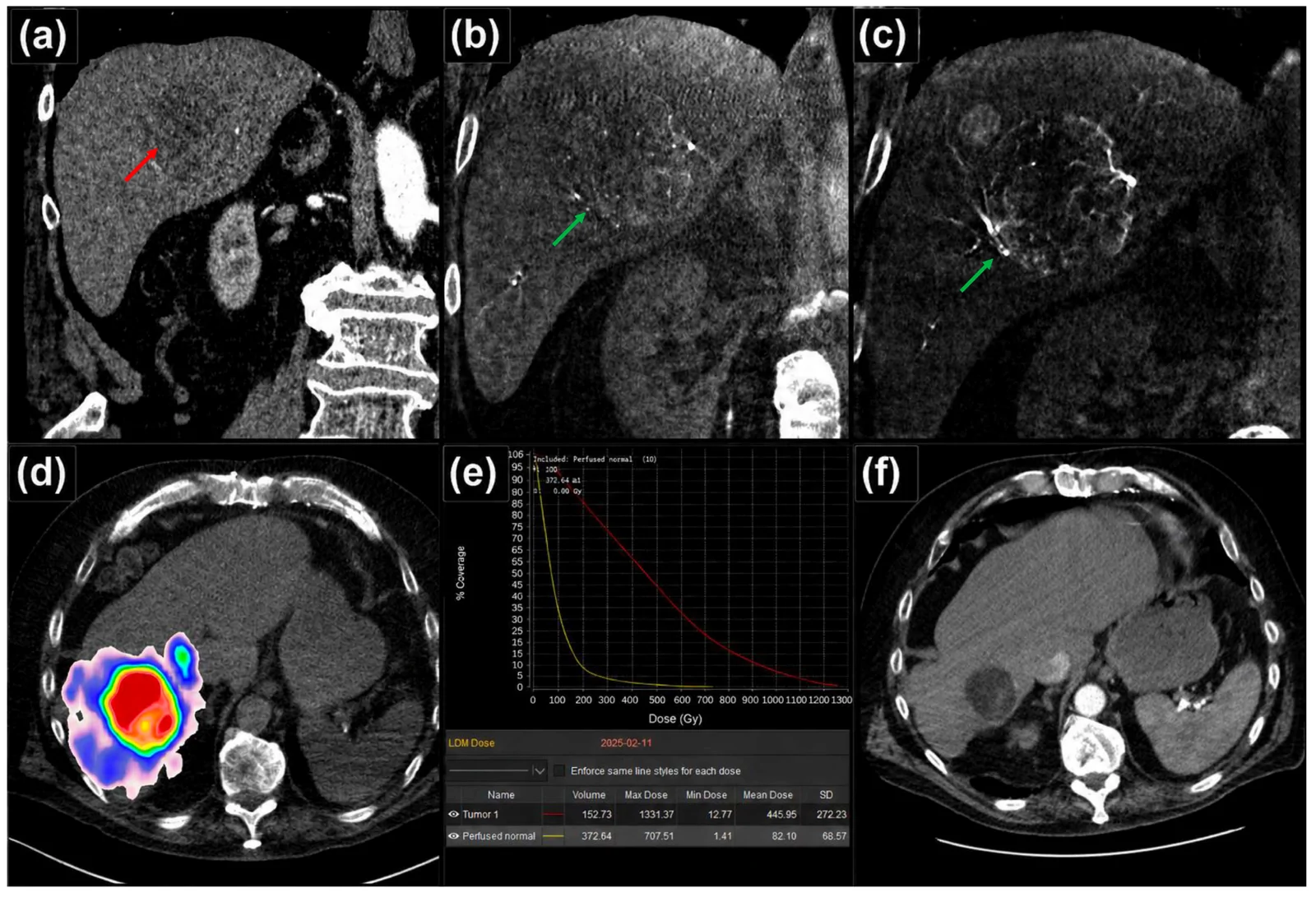
Pressure-enabled delivery catheter improves intratumoral perfusion of a hypovascular hepatocellular carcinoma prior to Y90 radioembolization. (**a**) Coronal contrast-enhanced CT scan in a 65-year-old man with MASH (metabolic dysfunction-associated steatohepatitis) cirrhosis shows a biopsy-proven, relatively hypovascular hepatocellular carcinoma (red arrow) in the right hepatic lobe. (**b**,**c**) Cone beam CT performed without and with a pressure-enabled delivery catheter during hepatic arteriography demonstrates marked improvement in intratumoral perfusion, including a conspicuously enhancing peritumoral nodule (green arrowhead). (**d**,**e**) Fused axial CT and dose-map image generated with MIM demonstrate significantly high dose delivered to the tumor as compared to the background liver (mean dose 446 vs. 82 Gy respectively) in the perfused area. (**f**) Coronal contrast-enhanced images obtained 12 months after treatment demonstrate complete response based on the mRECIST criteria. This is a Y-90 radioembolization (TARE) case shown as an example of personalized dosimetry in Section 3.1, and it also illustrates the pressure-enabled delivery catheter discussed in Section 4.5.

TARE is currently used for radiation segmentectomy, lobar disease control, bridging or downstaging therapy, and treatment of vascular invasion in selected patients. A single dose threshold does not apply to every treatment. The target dose depends on the microsphere platform, dosimetry model, tumor-to-normal uptake, perfused volume, previous therapies used, and liver reserve. Mapping angiography, assessment of lung shunting and extrahepatic arterial supply, prevention of nontarget deposition, and quantitative post-treatment imaging remain essential when performing TARE [1,14,15,16]. A representative example of selective Y-90 radioembolization including intraprocedural perfusion assessment and post-treatment dosimetry is shown in Figure 1.

### 3.2. TACE Optimization

**Figure 2:**
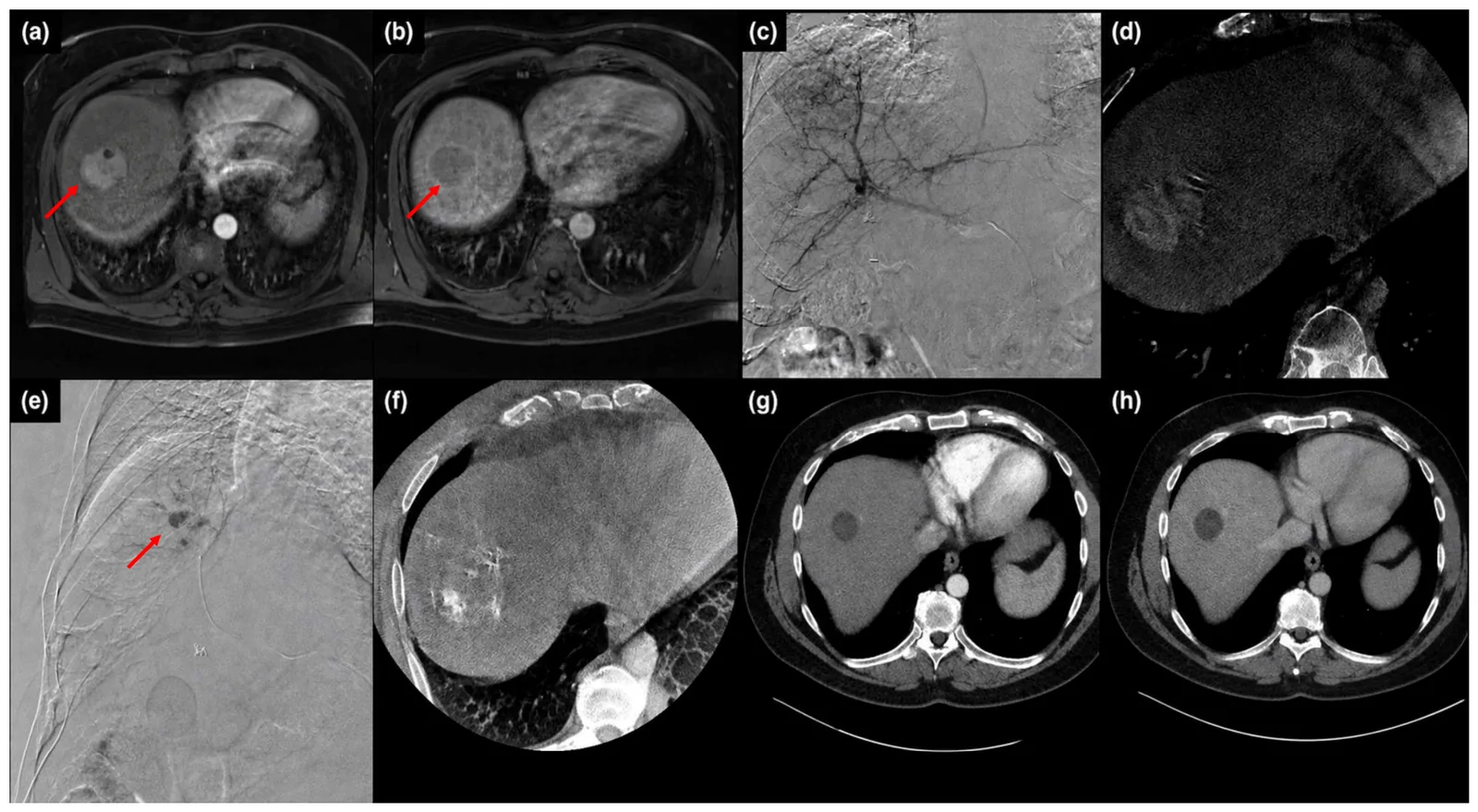
Segment VIII hepatocellular carcinoma treated with transarterial chemoembolization (TACE). (**a**) Arterial and (**b**) portal venous phase axial contrast-enhanced MR images in a 59-year-old woman show a hepatocellular carcinoma (red arrow) in segment VIII of the liver. (**c**) Nonselective hepatic arteriogram shows tumor neovascularity in the expected location within the liver. (**d**) Cone beam CT obtained after superselective catheterization of the segmental branch, prior to chemoembolization, confirms a hypervascular tumor in the expected location. (**e**) Post-embolization hepatic angiogram shows nonvisualization of tumor vascularity, with a vascular lakes (red arrow) in the treated area, consistent with retained embolic material. (**f**) Non-contrast cone beam CT obtained after DEB-TACE shows a near-complete rim of contrast with vascular lakes. (**g**) Arterial and (**h**) portal venous phase axial contrast-enhanced CT images obtained 6 months after DEB-TACE demonstrate complete response per mRECIST criteria.

Randomized trials established TACE as a treatment for selected cases of unresectable HCC [6,7]; however, contemporary practice differs from these original trials because the use of cone-beam computed tomography (CT), smaller microcatheters, superselective catheterization, and on-demand retreatment reduces treatment of uninvolved liver, in line with current technical standards of practice [17,18]. This selectivity is central to modern TACE procedures. A superselective TACE procedure using cone-beam CT for confirmation of tumor targeting is shown in Figure 2. Segmental or subsegmental treatment is preferred when feasible, whereas repeated lobar or bilobar embolization can precipitate hepatic decompensation. TACE is generally unsuitable for patients with severely impaired liver function, those with diffuse or infiltrative disease that cannot be treated selectively, and those for whom systemic therapy is more likely to provide timely disease control. Main portal vein obstruction, poor performance status, and uncontrolled extrahepatic disease require particular caution [1,2]. In addition, the specific technique used for TACE should be selected based on tumor vascularity and distribution, previous treatments used, biliary risk, operator experience, and institutional protocols. Whether to repeat TACE depends on how the tumor has responded. Recent consensus statements describe TACE refractoriness, meaning an inadequate objective response or intrahepatic progression of the treated lesions after two or more properly performed sessions, at times with a persistent rise in tumor markers. Once a patient reaches this point, the same statements advise a move to systemic therapy or another locoregional option [19].

### 3.3. Percutaneous Ablation: Modality Choice, Margins, and Navigation

Radiofrequency ablation (RFA) and microwave ablation (MWA) are curative-intent options for appropriately selected small HCCs, particularly tumors up to 3 cm. Local control declines as tumor size increases, and tumors larger than 3 cm may require multiple applicators, overlapping ablations, combination treatment, or use of another modality. Randomized studies comparing surgery with ablation support the use of ablation in selected patients, but the results are dependent on tumor location, treatment margin, and operator experience [2,5].

**Figure 3:**
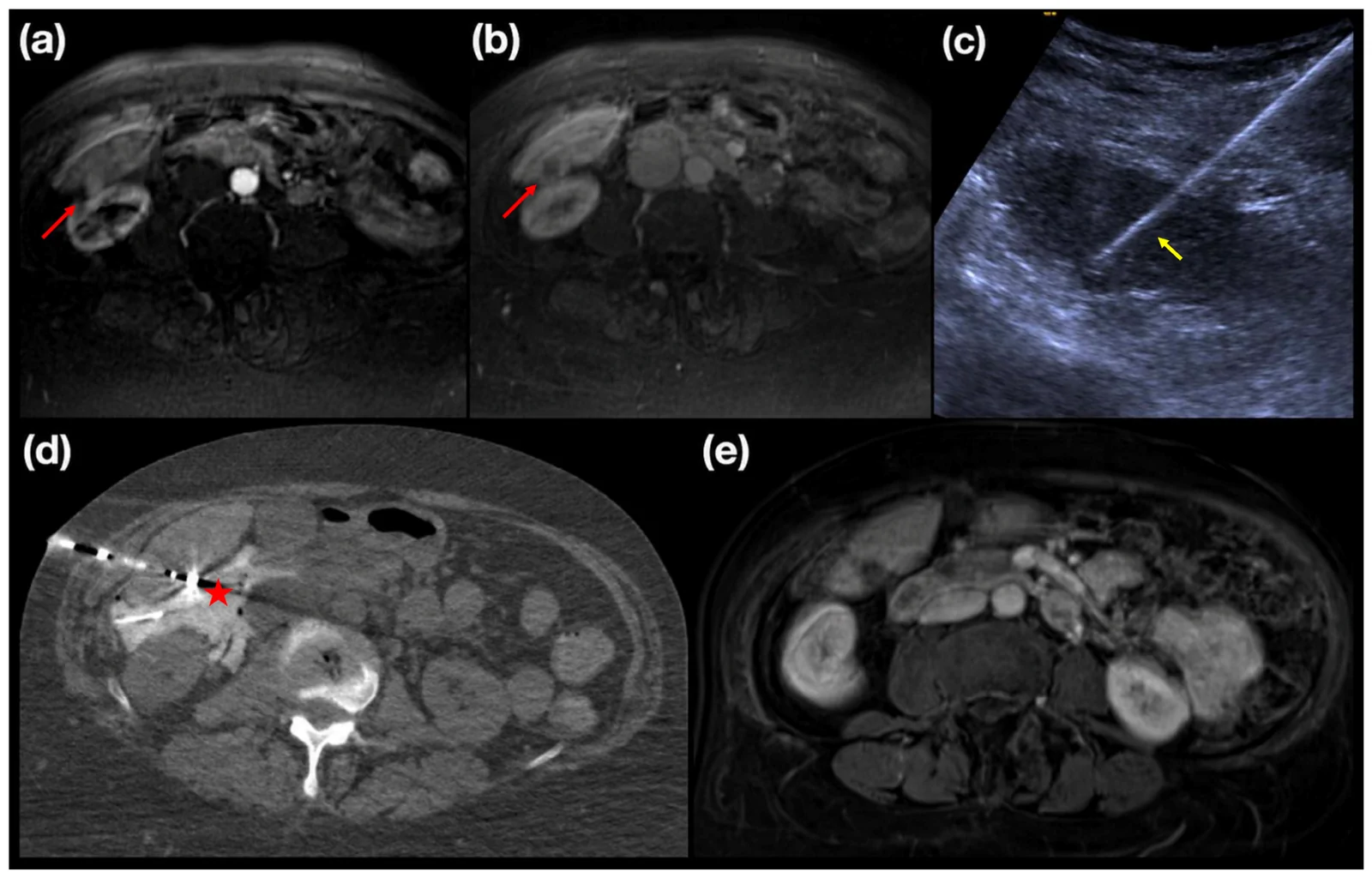
Percutaneous microwave ablation of an exophytic hepatocellular carcinoma. (**a**,**b**) Axial contrast-enhanced MR images in the arterial and portal venous phase in a 56-year-old woman show a 1.5 cm hepatocellular carcinoma (red arrow) in segment V of the right hepatic lobe. (**c**,**d**) Ultrasound and CT scan guided placement and thermal ablation of the microwave probe (yellow arrow) in the tumor with hydrodissection (red-colored star) displacing right kidney and hepatic flexure. Axial contrast-enhanced MR image shows the tumor with an exophytic component projecting beyond the hepatic contour, involving approximately 30% of its circumference. (**e**) Axial MR image obtained 6 months after ablation demonstrates complete response based on the mRECIST criteria.

MWA can produce higher intratumoral temperatures, larger ablation zones that can be created more quickly, and less sensitivity to convective cooling than conventional RFA. Meta-analyses suggest that MWA may offer modest advantages over RFA, in complete ablation or local tumor progression in some settings [20,21], but the studies have been heterogeneous, and major guidelines have not identified one thermal modality as universally superior. In general, RFA remains appropriate for small tumors in favorable locations, whereas MWA may be useful when a larger zone or shorter ablation time is needed [20,21]. Microwave ablation and radiofrequency ablation are used in different situations. Microwave ablation creates larger and faster ablation zones and is less sensitive to nearby blood flow, which makes it useful for tumors approaching or exceeding 3 cm and for lesions beside large vessels. Radiofrequency ablation produces a highly predictable and well-characterized zone, an advantage for small tumors in favorable locations and close to heat-sensitive structures. Trials have not shown a consistent overall-survival difference between them, so the choice follows tumor size and location, proximity to critical structures, and the operator’s experience. An example of percutaneous MWA with hydrodissection to protect adjacent structures is shown in Figure 3.

Recent device development has focused on achieving more predictable ablation geometry and adequate margins. Multielectrode or no-touch RFA systems create overlapping treatment zones around the tumor, and prospective studies have reported low local progression for small HCCs when coverage is adequate [22]. Thermosphere-oriented MWA systems have been designed to reduce elongation of the ablation zone [23]. Pulsed MWA is being studied for high-risk locations, but the current evidence is retrospective and does not establish superiority over well-planned conventional MWA [24].

With ablation, margin is generally more important than the generator used to create it. When anatomy permits, a circumferential margin of approximately 5 to 10 mm is sought because an insufficient minimal margin is a major determinant of local recurrence. To this end, fusion imaging can make inconspicuous tumors targetable, stereotactic navigation can improve reproducibility, and intraprocedural image registration can be used to check applicator position and treatment coverage [25]. For instance, the randomized phase 2 COVER-ALL trial compared software-assisted registration and segmentation with visual margin assessment [26]. Software assistance increased the minimal ablation margin and the proportion of tumors that met the prespecified margin without a clear increase in adverse events. The trial was not powered to assess local control or survival, but it demonstrated that a modifiable technical endpoint linked to recurrence can be measured and improved during the procedure. A 2026 international multisociety Delphi consensus, endorsed by the European Society of Surgical Oncology together with the Cardiovascular and Interventional Radiological Society of Europe and the Society of Interventional Oncology, went further and recommended that the minimal ablative margin be measured in three dimensions on contrast-enhanced CT or MRI and documented for every treated tumor, and that it be confirmed during the procedure with ablation-confirmation software where this is available [26,27].

### 3.4. Integration of Locoregional and Systemic Therapy

Locoregional treatment can release tumor antigens, danger-associated molecular patterns, and inflammatory cytokines, thus providing a biologic rationale for combination with immune checkpoint inhibition. However, mechanistic rationale alone does not establish clinical benefit, as the immune effect from locoregional therapy is likely to vary with modality, completeness of treatment, tumor burden, background liver disease, and therapy timing [28].

The strongest randomized evidence for combining locoregional and systemic treatment currently comes from TACE-based regimens. In EMERALD-1, adding durvalumab plus bevacizumab to TACE prolonged progression-free survival compared with TACE alone, whereas durvalumab without bevacizumab did not produce the same benefit [29]. LEAP-012 likewise showed longer progression-free survival when lenvatinib plus pembrolizumab was administered with TACE [30]. In EMERALD-1 (616 patients), median progression-free survival improved from 8.2 to 15.0 months (hazard ratio [HR] 0.77, 95% confidence interval [CI] 0.61–0.98). LEAP-012 (480 patients) showed a comparable gain, from 10.0 to 14.6 months (HR 0.66, 95% CI 0.51–0.84). Both trials enrolled patients with unresectable, nonmetastatic HCC, preserved liver function, and suitability for embolization [29,30]. Their findings therefore support combination treatment for selected patients rather than its routine use across the entire intermediate-stage population, and the survival data support this cautious view, because the final overall survival result in EMERALD-1 and the interim result in LEAP-012 both fell short of statistical significance, so the progression-free survival gain is established while an overall survival gain is not yet confirmed [29,30].

Evidence supporting the use of TARE combined with immune checkpoint inhibition has mainly been reported in retrospective or single-arm studies. Reported response and survival rates from these studies are encouraging, but the selection bias is substantial because patients who receive both treatments often have preserved liver function and access to specialized multidisciplinary care. Randomized trials are needed to determine whether TARE adds benefit to modern systemic therapy, which patients are most likely to benefit, and whether treatment should be concurrent or sequential [9,31,32].

The initial IMbrave050 study analysis reported improved recurrence-free survival with adjuvant atezolizumab plus bevacizumab after resection or ablation in patients with high-risk HCC [33]. However, the results for overall survival were immature, and the study combined patients treated with surgery or ablation into one group. Thus, these findings did not establish support for the routine use of adjuvant atezolizumab plus bevacizumab after curative ablation. An updated analysis, reported after a median follow-up of 35.1 months, gave a different result. The early recurrence-free survival advantage (HR 0.72, 95% CI 0.53–0.98) narrowed with longer follow-up, giving an updated hazard ratio of 0.90 (95% CI 0.72–1.12) and median recurrence-free survival of 33.2 months with treatment and 36.0 months with surveillance. Overall survival remained immature (HR 1.26, 95% CI 0.85–1.87), and the investigators concluded that the balance of benefit and risk no longer justifies routine adjuvant atezolizumab plus bevacizumab after curative-intent resection or ablation [34].

Combination strategies raise practical questions about which locoregional modality best complements a systemic regimen, whether treatment should be concurrent or sequential, and how long antiangiogenic agents should be withheld around invasive procedures. Bleeding, hypertension, hepatic decompensation, cumulative treatment burden, and the distinction between immune-related hepatitis and post-treatment liver injury must be considered. The definition of TACE failure and the risk of delaying effective systemic therapy are also important, particularly when repeat embolization is contemplated. In addition, exposure to immunotherapy may affect transplant candidacy. These regimens should therefore be selected through coordinated multidisciplinary review rather than treated as a universal replacement for selective locoregional therapy or upfront systemic therapy [9,29,30].

### 3.5. Comparative and Combination Locoregional Strategies

Direct comparisons between TARE and TACE are still few. The phase 2 TRACE randomized trial compared Y-90 radioembolization with drug-eluting-bead TACE in BCLC stage A and B disease and favored radioembolization for both tumor control and survival, with a median time to progression of 17.1 versus 9.5 months (HR 0.36, 95% CI 0.18–0.70) and median overall survival of 30.2 versus 15.6 months (HR 0.48, 95% CI 0.28–0.82) [35]. The wider randomized evidence is more mixed, and pooled analyses tend to show similar overall survival with a gentler adverse-event profile for TARE. Combination treatment is a second theme. A meta-analysis of 21 studies and 3413 patients found that TACE plus radiofrequency ablation improved overall survival over TACE alone (HR 0.62, 95% CI 0.55–0.71) and over ablation alone (HR 0.63, 95% CI 0.53–0.75), and the advantage over ablation alone was clearest for tumors larger than 3 cm [36]. In each case, the decision rests on tumor size, number, location, and liver reserve, and it belongs in a multidisciplinary discussion. Table 2 gathers the design, size, primary-endpoint effect sizes, and follow-up for the pivotal studies discussed here.

## 4. Emerging and Investigational Technologies

Most technologies discussed in this section have not been compared with current standard treatments in HCC-specific randomized trials. The available studies must be interpreted according to whether they report technical feasibility, local control, or survival outcomes.

### 4.1. Histotripsy

**Figure 4:**
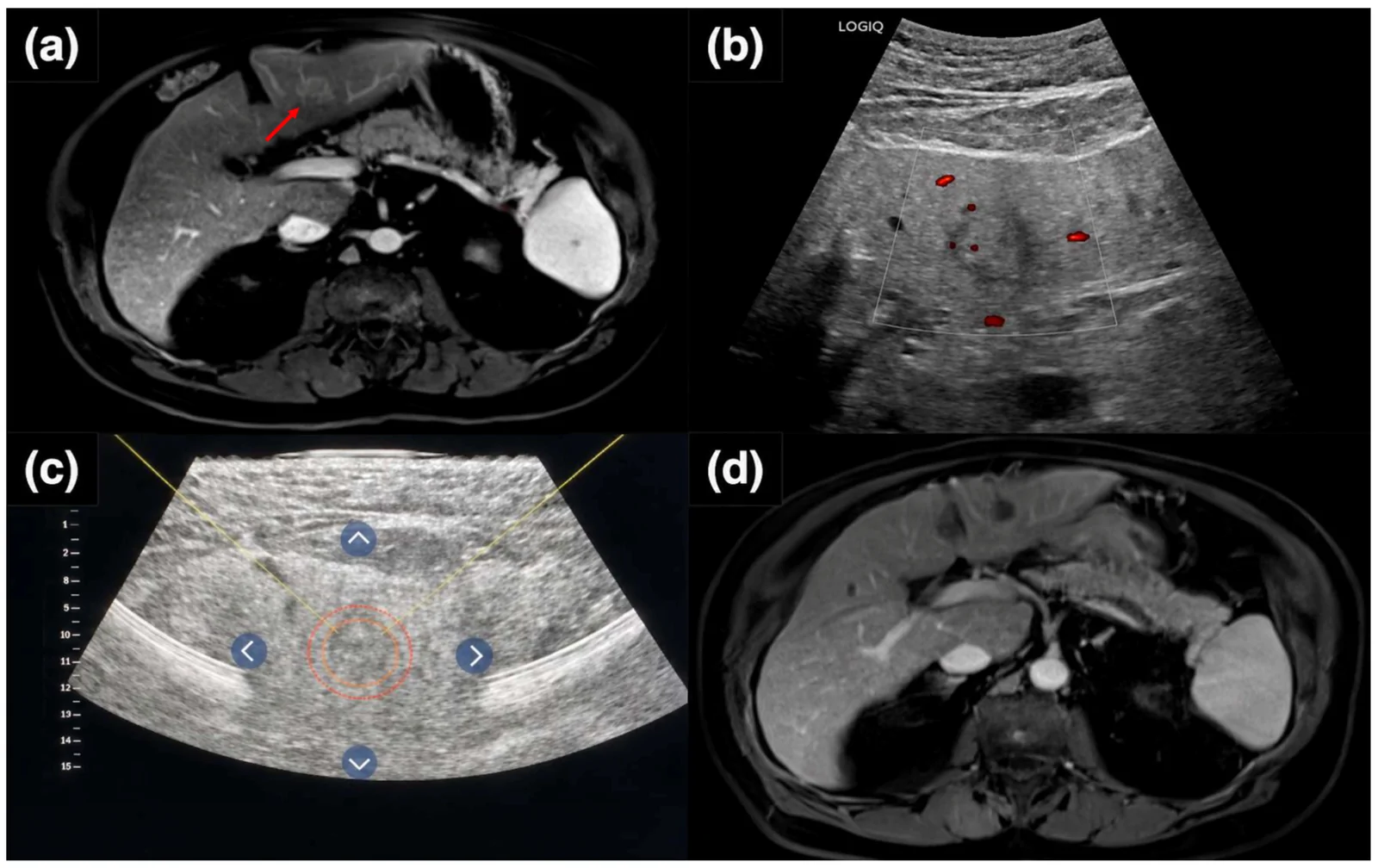
Histotripsy of the hepatocellular carcinoma in the left hepatic lobe. (**a**) Axial contrast-enhanced image in a 66-year-old man shows the hepatocellular carcinoma (red arrow) in the left hepatic lobe. (**b**,**c**) Pre- and intraprocedural ultrasound images demonstrating visualization of the tumor and treatment planning during histotripsy respectively. (**d**) Axial contrast-enhanced image obtained 6 months after treatment demonstrates complete response, with no residual or recurrent enhancing tumor, in accordance with mRECIST criteria.

Histotripsy is a nonthermal, nonionizing technique that uses focused ultrasound pulses to generate acoustic cavitation and mechanically fractionate tissue. The noninvasive delivery, real-time ultrasound visualization, and relative preservation of collagen-rich structures associated with this technique make histotripsy an attractive option for the treatment of selected liver tumors as shown in Figure 4. However, clinical evidence supporting the use of histotripsy for HCC is still limited. The THERESA and HOPE4LIVER studies reported high technical success rates and an acceptable short-term safety profile when histotripsy was used in mixed cohorts of patients with primary and metastatic liver tumors [37,40]. The evidence for histotripsy has grown over the past few years. The Edison system received U.S. FDA De Novo authorization for liver tumors in October 2023, and the one-year HOPE4LIVER update, which included 47 patients and 19 with HCC, reported one-year local control of 63.4% on primary assessment and 90% on post hoc review, together with one-year overall survival of 73.3% in the HCC subgroup [38]. These results come from single-arm cohorts, so they show early efficacy in HCC without yet establishing how histotripsy compares with the established options. For now, it remains an investigational choice alongside ablation, TARE, surgery, and stereotactic radiation.

Current challenges associated with histotripsy include the need for an adequate acoustic window, the presence of rib or lung interference, the presence of bowel gas or respiratory motion, depth of the tumor, limitations associated with patient body habitus, and restricted access to parts of the hepatic dome. The use of aberration-correction methods may expand the treatable volume, but these techniques remain developmental. Immunostimulatory and abscopal effects have been reported in animal models; clinical confirmation in patients with HCC is required [41,42].

### 4.2. Electric-Field Ablation and Electrochemotherapy

Irreversible electroporation (IRE) uses high-voltage electrical pulses to create permanent membrane pores and predominantly nonthermal cell death. Because the extracellular matrix is relatively preserved and treatment is less dependent on heat transfer, IRE is most useful for small tumors near major vessels or central bile ducts that are poor candidates for thermal ablation. The procedure requires the use of general anesthesia, cardiac synchronization, precise placement of multiple parallel electrodes, and experience with electric-field planning [43]. Clinical results with this technique have been variable. An early series reported local progression rates higher than those expected after technically successful thermal ablation in favorable locations [43], whereas propensity-matched data suggested that IRE may perform well in carefully selected perivascular tumors [44]. No randomized study has shown that IRE is superior to RFA, MWA, TARE, or stereotactic radiation.

Nanosecond pulsed electric-field ablation uses shorter pulses intended to affect intracellular organelles as well as the cell membrane. A prospective, multicenter study of 192 patients with 217 HCCs in high-risk locations, next to major vessels, bile ducts, the gallbladder, the gastrointestinal tract, or the diaphragm, reported a 99.5% technical success rate and complete ablation of 91.7% of tumors. Local tumor progression at 1, 2, and 3 years was 9.8%, 13.8%, and 15.7%, and recurrence-free survival at the same time points was 72.2%, 51.7%, and 43.5% over a median follow-up of 33.5 months [39]. This cohort is larger and has longer follow-up than earlier reports, and it supports a clinical role for nanosecond pulsed-field ablation, with comparative trials and external validation still needed. The use of immune priming is supported by preclinical studies and remains an investigational rationale rather than a proven clinical advantage [45].

Electrochemotherapy is a technique that combines reversible electroporation with bleomycin or cisplatin to increase intracellular drug delivery. Prospective studies of this technique in patients with HCC have been small, have frequently employed the technique intraoperatively, and have been concentrated in only a few centers [46,47]. Despite these limitations, the findings from these series have been encouraging, including supporting the treatment of portal vein tumor thrombus in selected patients, but the current evidence is insufficient for routine first-line use. At present, electrochemotherapy is best considered an investigational or salvage option for carefully selected patients.

### 4.3. Cryoablation

Cryoablation creates an image-visible ice ball and may be useful for tumors near vessels, the diaphragm, or pain-sensitive structures. Modern argon–helium systems have reduced, but not eliminated, the historical risk of cryoshock; a 2024 meta-analysis found that this complication is rare [48]. Comparative HCC data remain less mature than for RFA and MWA, and major guidelines do not routinely prefer cryoablation [1,2]. The factors of perfusion, freeze-zone geometry, and collateral injury remain clinically relevant near vessels and bile ducts [49].

Cryoablation preserves more native protein structure than heat-based ablation and may produce a different immune response. Reviews and murine HCC models support this hypothesis [50,51], but clinical evidence for superior systemic immunity or an abscopal effect is currently insufficient. Prospective studies assessing the combination of cryoablation with checkpoint inhibition are needed.

### 4.4. High-Intensity Focused Ultrasound

High-intensity focused ultrasound (HIFU) is noninvasive but thermally ablative. Treatment with this technique is limited by the acoustic window, respiratory motion, rib and lung interference, procedure duration, and perfusion-mediated cooling near large vessels. Most evidence regarding the use of HIFU for HCC comes from single-center or regional studies, and HIFU is not a standard recommendation in major North American or European HCC guidelines [1,2]. Meta-analyses of TACE plus HIFU suggest a possible benefit, but heterogeneity, older comparators, and variable study quality limit confidence in these findings [52,53].

Focused ultrasound can also be used to trigger drug delivery rather than to ablate tissue. In the phase 1 TARDOX study, mild focused-ultrasound hyperthermia increased intratumoral doxorubicin release from thermosensitive liposomes in liver tumors [54]. However, this was a proof-of-concept study and did not establish clinical benefit for HCC.

### 4.5. Next-Generation Radioembolization, Embolics, and Regional Drug Delivery

Several platforms are also being developed to improve visualization and verification of radioembolization. Radiopaque Y-90 microspheres can be seen on CT, which may allow for direct assessment of microsphere distribution and post-treatment dosimetry. First-in-human data regarding this technique are encouraging [55], but comparative evidence is not yet available. Holmium-166 microspheres emit beta particles and gamma photons and are paramagnetic, allowing quantitative assessment with single-photon emission CT and magnetic resonance imaging. European experience and early adaptive-dosimetry studies support the feasibility of this technique, although the HCC evidence base remains substantially larger for Y-90 treatment [56,57,58].

Pressure-enabled delivery catheters are intended to alter intra-arterial pressure gradients and increase penetration of embolic material. Porcine research has shown greater microsphere deposition with these catheters than with conventional end-hole catheter delivery [59], but animal biodistribution does not establish improved tumor control or survival The potential effect of pressure-enabled delivery on intratumoral perfusion during Y-90 radioembolization is illustrated in Figure 1. Clinical studies are needed to evaluate delivered dose, nontarget deposition, toxicity, and oncologic outcomes with this technique.

Liquid hydrogel embolics can penetrate distal tumor vasculature and conform to vessel geometry. A first-in-human study of the liquid embolic agent Embrace demonstrated technical feasibility in a small mixed cohort, but it did not establish clinical superiority or mature data for the treatment of HCC [60]. Transarterial chemo-immuno-embolization, nanoparticle carriers, and immune-modulating drug-eluting beads are additional early platforms currently being studied [61,62,63]. Further development will require reproducible manufacturing, pharmacokinetic and drug-release studies, and trials assessing comparative clinical endpoints.

### 4.6. Robotic Assistance, Augmented Reality, and Artificial Intelligence

Robotic and stereotactic systems are designed to translate a planned three-dimensional trajectory into accurate applicator placement. Randomized and prospective studies have reported better targeting precision, fewer repositioning attempts, and lower operator or patient radiation exposure with these systems in some settings [64,65,66]. These are relevant procedural endpoints, particularly for deep or difficult-to-access tumors, but the studies included heterogeneous liver tumors and did not demonstrate improved HCC-specific local control or survival.

Future systems will likely combine automated segmentation, deformable registration, respiratory-motion compensation, trajectory planning, robotic needle placement, and immediate margin verification. Augmented-reality platforms may improve spatial understanding, but registration error and human-factors testing remain important. Successful clinical adoption of these techniques will require consideration of factors such as prospective oncologic outcomes, workflow efficiency, training requirements, cybersecurity, and cost-effectiveness [26,67].

Artificial intelligence contributes to interventional oncology mainly as a decision aid. In HCC, its current applications include patient selection, prediction of treatment response, segmentation of tumors and vessels, image guidance and registration, margin assessment, and outcome prediction. The most developed of these is prediction of TACE response. A systematic review and meta-analysis of radiomic and deep learning models found a pooled area under the receiver-operating-characteristic curve of about 0.89 on internal validation and 0.81 on external validation, with no significant difference between the two [68]. Good discrimination of this kind shows that the models rank patients well, though it does not by itself show that they change treatment decisions or outcomes. Most studies so far are retrospective and single-center, and wider use will depend on external validation, interpretability, integration into workflow, and prospective evidence of patient benefit.

Table 3 summarizes the current role and strength of evidence for the modalities discussed in this review.

## 5. Implementation, Equity, and Research Priorities

Access to advanced interventional technology is uneven. Personalized radioembolization requires nuclear medicine infrastructure and dosimetry expertise; stereotactic or robotic ablation requires capital equipment and trained teams; and histotripsy is currently available only in selected centers. Additionally, results from high-volume tertiary centers may not be reproducible in all practice settings. Comparative studies should therefore include data regarding operator experience, learning curves, treatment access, travel burden, reimbursement, and implementation requirements.

Cost and resource use differ widely across these modalities, and they matter most where budgets are tight. Percutaneous thermal ablation is usually the least resource-intensive curative-intent option, since it needs only modest disposables and imaging, while TACE carries an intermediate cost and TARE with Y-90 is the most expensive per procedure because it depends on microspheres, nuclear-medicine infrastructure, and dosimetry expertise. The newer platforms, including histotripsy and robotic or stereotactic navigation systems, require major capital investment, and combining systemic agents with locoregional therapy adds drug costs and monitoring. Because cost-effectiveness shifts with disease stage, local pricing, and reimbursement, economic endpoints deserve to be reported next to clinical outcomes, and each technology should be judged against the capacity of the setting where it will be used, especially in lower-resource settings.

Future trials should also stratify patients by tumor burden, liver function, transplant candidacy, and previous therapy and should use standardized response criteria and toxicity reporting. Ablation studies should report local tumor progression and minimal margin. Transarterial studies should report embolization selectivity and, for radioembolization, tumor-absorbed and normal-liver-absorbed dose. Across modalities, data regarding liver function preservation, patient-reported outcomes, time to systemic therapy, transplant rate, overall survival, and cost-effectiveness should be included.

Studies should avoid combining HCC with metastatic liver tumors without subgroup reporting. In addition, retrospective cohorts should not be pooled with randomized trials as though they were equivalent, and survival benefit should not be inferred from radiologic response alone. Single-center technical success should not be assumed to imply superiority of a treatment. Prospective registries and pragmatic comparative trials may be useful when rapid device iteration makes conventional trial timelines difficult.

## 6. Conclusions

Current evidence supports the use of personalized TARE dosimetry and radiation segmentectomy, selective TACE with reassessment before repeat treatment, and margin-controlled percutaneous ablation supported by fusion imaging and software-assisted assessment in patients with HCC. Phase 3 trials of TACE combined with systemic therapy have shown a progression-free survival benefit in selected patients with intermediate-stage disease, but mature overall survival, sequencing, safety, and quality of life data are still needed.

Histotripsy, pulsed electric-field treatment, electrochemotherapy, cryoablation, HIFU, imageable or alternative microspheres, new embolics, robotics, and artificial intelligence remain at different stages of development. Their clinical role should be determined by HCC-specific prospective studies focusing on patient-centered outcomes, including durable tumor control, preserved liver function, eligibility for curative treatment, quality of life, and survival. Multidisciplinary review is necessary when these therapies are integrated into clinical practice.

## Figures and Tables

**Table 1:** Position of interventional oncology within the BCLC staging framework for HCC.

BCLC Stage	Standard or First-Line Treatment	Role of Interventional Oncology	Emerging Options
Very early and early (0 to A)	Resection, transplantation, ablation	Thermal ablation for cure; radiation segmentectomy; TARE or TACE to bridge to transplant	Histotripsy; nanosecond pulsed-field ablation; margin-confirmation software
Intermediate (B)	TACE, with systemic therapy in selected patients	Selective or drug-eluting-bead TACE; TARE for lobar control; TACE combined with ablation	TACE with immunotherapy (EMERALD-1, LEAP-012); personalized TARE dosimetry
Advanced (C)	Systemic therapy such as atezolizumab plus bevacizumab or durvalumab plus tremelimumab	TARE for selected portal-vein invasion; locoregional and systemic combinations	Trials that sequence locoregional therapy with immunotherapy
Terminal (D)	Best supportive care	None	None

**Table 2:** Key clinical studies of locoregional and combination therapy for HCC discussed in this review.

Study (Design)	N	Primary or Key Endpoint Result (Effect Size, 95% CI)	Follow-Up
DOSISPHERE-01 (RCT phase 2; personalized vs. standard TARE dosimetry) [12]	60	Index-lesion objective response 71% vs. 36%; median OS 26.6 vs. 10.7 months (HR 0.42, 95% CI 0.21–0.83)	3-month primary endpoint
SARAH (RCT phase 3; resin-microsphere TARE vs. sorafenib) [11]	459	Median OS 8.0 vs. 9.9 months (HR 1.15, 95% CI 0.94–1.41); no OS benefit	Median FU 27.9 months
LEGACY (retrospective cohort; radiation segmentectomy) [15]	162	Best objective response 88.3%; 3-year OS 86.6%	Median FU 29.9 months
RASER (prospective single-arm; radiation segmentectomy) [14]	29	High complete-response rate and durable local control (single-arm)	Not reported
COVER-ALL (RCT phase 2; software vs. visual margin assessment) [26]	Not reported	Larger minimal ablative margin and a higher proportion meeting the target margin; not powered for local control	Not reported
TRACE (RCT phase 2; Y-90 TARE vs. DEB-TACE) [35]	72	Time to progression 17.1 vs. 9.5 months (HR 0.36, 0.18–0.70); OS 30.2 vs. 15.6 months (HR 0.48, 0.28–0.82)	Stopped early at interim
EMERALD-1 (RCT phase 3; TACE with or without durvalumab and bevacizumab) [29]	616	PFS 15.0 vs. 8.2 months (HR 0.77, 0.61–0.98); final OS not significant	Median FU 27.9 mo (PFS)
LEAP-012 (RCT phase 3; TACE with or without lenvatinib and pembrolizumab) [30]	480	PFS 14.6 vs. 10.0 months (HR 0.66, 0.51–0.84); interim OS not significant (HR 0.80, 0.57–1.11)	Median FU 25.6 months
IMbrave050 (RCT phase 3; adjuvant atezolizumab and bevacizumab vs. surveillance) [33,34]	668	Updated RFS HR 0.90 (0.72–1.12); early benefit not sustained; OS immature (HR 1.26, 0.85–1.87)	Median FU 35.1 months
HOPE4LIVER (single-arm pivotal; histotripsy) [37,38]	47 (19 HCC)	1-year local control 63.4% (90% post hoc); 1-year OS 73.3% in HCC	1 year
nsPEF, Xu et al. (prospective multicenter; high-risk locations) [39]	192 (217 tumors)	Complete ablation 91.7%; 3-year local tumor progression 15.7%; 3-year RFS 43.5%	Median FU 33.5 months
TACE plus RFA meta-analysis (21 studies) [36]	3413	OS vs. TACE alone HR 0.62 (0.55–0.71); OS vs. RFA alone HR 0.63 (0.53–0.75)	Pooled

**Table 3:** Current role, strength of evidence, and key studies for locoregional and emerging modalities in HCC.

Modality	Current Role and Intent	Strength of Evidence	Key Studies
Personalized TARE dosimetry and radiation segmentectomy	Curative intent for segmentectomy; disease control and bridging or downstaging otherwise	Randomized dosimetry data with prospective and cohort support	DOSISPHERE-01 [12]; LEGACY [15]; RASER [14]
Selective or drug-eluting-bead TACE	Standard for intermediate-stage disease and for bridging	Randomized and established, with technical consensus	[6,7,17,27]
Thermal ablation (RFA or MWA) with margin confirmation	Curative intent for early HCC up to 3 cm	Randomized against surgery and for margin, with consensus	[5,20,21,26,27]
TACE with systemic therapy	Selected intermediate-stage disease	Phase 3, with a PFS benefit and no established OS benefit	EMERALD-1 [29]; LEAP-012 [30]
TARE compared with TACE, and TACE with ablation	Comparative and combination strategies	Randomized (TRACE) with meta-analysis	TRACE [35]; Jiang [36]
Histotripsy	Investigational noninvasive ablation	Single-arm pivotal	THERESA [40]; HOPE4LIVER [37,38]
Nanosecond pulsed-field ablation	Emerging clinical use in high-risk locations	Prospective multicenter	Xu et al. [39]
IRE and electrochemotherapy	Investigational or salvage for perivascular disease	Retrospective and small prospective	[43,44,46,47,69]
Cryoablation	Selected use, not guideline-preferred	Retrospective and meta-analysis	[48,49,50,51]
High-intensity focused ultrasound	Not a standard recommendation	Single-center and meta-analysis	[52,53]
Next-generation radioembolics and embolics	Investigational	First-in-human and preclinical	[55,56,57,58,59,60,61,62,63]
Robotics and augmented reality	Procedural adjunct	Randomized for targeting endpoints	[64,65,66,67]
Artificial intelligence	Supportive, for selection, response prediction, segmentation, and guidance	Retrospective with systematic review and meta-analysis, including external validation	[68]
CBCT	Cone-beam computed tomography		
Y-90	Yttrium-90		
DEB-TACE	Drug-eluting bead transarterial chemoembolization		
mRECIST	Modified Response Evaluation Criteria in Solid Tumors		
ALBI	Albumin-bilirubin		
MASH	Metabolic dysfunction-associated steatohepatitis		
SPECT	Single-photon emission computed tomography		
MRI	Magnetic resonance imaging		
PFS	Progression-free survival		
OS	Overall survival		
ORR	Objective response rate		
AI	Artificial intelligence		

## Data Availability

No new data were created or analyzed in this study. Data sharing is not applicable to this article.

## Abbreviations

The following abbreviations are used in this manuscript:

HCC	Hepatocellular carcinoma
TACE	Transarterial chemoembolization
TARE	Transarterial radioembolization
CT	Computed tomography
RFA	Radiofrequency ablation
MWA	Microwave ablation
IRE	Irreversible electroporation
HIFU	High-intensity focused ultrasound

## References

[1] Reig M., Forner A., Rimola J., Ferrer-Fàbrega J., Burrel M., Garcia-Criado Á., Kelley R.K., Galle P.R., Mazzaferro V., Salem R. (2022). BCLC strategy for prognosis prediction and treatment recommendation: The 2022 update. J. Hepatol..

[2] Singal A.G., Llovet J.M., Yarchoan M., Mehta N., Heimbach J.K., Dawson L.A., Jou J.H., Kulik L.M., Agopian V.G., Marrero J.A. (2023). AASLD Practice Guidance on prevention, diagnosis, and treatment of hepatocellular carcinoma. Hepatology.

[3] Vogel A., Meyer T., Sapisochin G., Salem R., Saborowski A. (2022). Hepatocellular carcinoma. Lancet.

[4] Choi D.T., Kum H.C., Park S., Ohsfeldt R.L., Shen Y., Parikh N.D., Singal A.G. (2019). Hepatocellular Carcinoma Screening Is Associated With Increased Survival of Patients with Cirrhosis. Clin. Gastroenterol. Hepatol..

[5] Kawaguchi Y., Hasegawa K., Kashiwabara K., Okamura Y., Kurosaki M., Kudo M., Shimada M., Yamanaka N., Inomata M., Yamashita T. (2025). Surgery Versus Ablation for Hepatocellular Carcinoma: A Randomized Controlled Trial (SURF-RCT Trial) and a Nonrandomized Prospective Observational Trial (SURF-Cohort Trial). J. Clin. Oncol..

[6] Llovet J.M., Real M.I., Montana X., Planas R., Coll S., Aponte J., Ayuso C., Sala M., Muchart J., Sola R. (2002). Arterial embolisation or chemoembolisation versus symptomatic treatment in patients with unresectable hepatocellular carcinoma: A randomised controlled trial. Lancet.

[7] Lo C.M., Ngan H., Tso W.K., Liu C.L., Lam C.M., Poon R.T., Fan S.T., Wong J. (2002). Randomized controlled trial of transarterial lipiodol chemoembolization for unresectable hepatocellular carcinoma. Hepatology.

[8] Guan M.C., Ding Q., Zhao Q., Li N., Zhang R.X., Zhang S.Y., Wang J., Zhu H. (2025). Albumin-bilirubin grade as an alternative to Child-Pugh class for evaluating liver function within staging systems for hepatocellular carcinoma. Discov. Oncol..

[9] Thornton L.M., Abi-Jaoudeh N., Lim H.J., Malagari K., Spieler B.O., Kudo M., Finn R.S., Lencioni R., White S.B., Kokabi N. (2024). Combination and Optimal Sequencing of Systemic and Locoregional Therapies in Hepatocellular Carcinoma: Proceedings from the Society of Interventional Radiology Foundation Research Consensus Panel. J. Vasc. Interv. Radiol..

[10] Llovet J.M., Mauro E., Rimassa L., Mazzaferro V., Pinter M., Kelley R.K., Chan S.L., Kulik L., Pillai A., Roberts L. (2026). Trial design and end points in hepatocellular carcinoma: An EASL-AASLD-ILCA consensus statement. Nat. Rev. Clin. Oncol..

[11] Vilgrain V., Pereira H., Assenat E., Guiu B., Ilonca A.D., Pageaux G.P., Sibert A., Bouattour M., Lebtahi R., Allaham W. (2017). Efficacy and safety of selective internal radiotherapy with yttrium-90 resin microspheres compared with sorafenib in locally advanced and inoperable hepatocellular carcinoma (SARAH): An open-label randomised controlled phase 3 trial. Lancet Oncol..

[12] Garin E., Tselikas L., Guiu B., Chalaye J., Edeline J., de Baere T., Assenat E., Tacher V., Robert C., Terroir-Cassou-Mounat M. (2021). Personalised versus standard dosimetry approach of selective internal radiation therapy in patients with locally advanced hepatocellular carcinoma (DOSISPHERE-01): A randomised, multicentre, open-label phase 2 trial. Lancet Gastroenterol. Hepatol..

[13] Hermann A.L., Dieudonne A., Ronot M., Sanchez M., Pereira H., Chatellier G., Garin E., Castera L., Lebtahi R., Vilgrain V. (2020). Relationship of Tumor Radiation-absorbed Dose to Survival and Response in Hepatocellular Carcinoma Treated with Transarterial Radioembolization with (90)Y in the SARAH Study. Radiology.

[14] Kim E., Sher A., Abboud G., Schwartz M., Facciuto M., Tabrizian P., Knesaurek K., Fischman A., Patel R., Nowakowski S. (2022). Radiation segmentectomy for curative intent of unresectable very early to early stage hepatocellular carcinoma (RASER): A single-centre, single-arm study. Lancet Gastroenterol. Hepatol..

[15] Salem R., Johnson G.E., Kim E., Riaz A., Bishay V., Boucher E., Fowers K., Lewandowski R., Padia S.A. (2021). Yttrium-90 Radioembolization for the Treatment of Solitary, Unresectable HCC: The LEGACY Study. Hepatology.

[16] Busse N.C., Al-Ghazi M., Abi-Jaoudeh N., Alvarez D., Ayan A.S., Chen E., Chuong M.D., Dezarn W.A., Enger S.A., Graves S.A. (2024). AAPM Medical Physics Practice Guideline 14.a: Yttrium-90 microsphere radioembolization. J. Appl. Clin. Med. Phys..

[17] Lammer J., Malagari K., Vogl T., Pilleul F., Denys A., Watkinson A., Pitton M., Sergent G., Pfammatter T., Terraz S. (2010). Prospective randomized study of doxorubicin-eluting-bead embolization in the treatment of hepatocellular carcinoma: Results of the PRECISION V study. Cardiovasc. Interv. Radiol..

[18] Lucatelli P., Burrel M., Guiu B., de Rubeis G., van Delden O., Helmberger T. (2021). CIRSE Standards of Practice on Hepatic Transarterial Chemoembolisation. Cardiovasc. Interv. Radiol..

[19] Zhong B., Zhang S., Zhu H., Wang W., Ni C., Clinical Guidelines Committee of Chinese College of Interventionalists (2024). Transarterial chemoembolization refractoriness in hepatocellular carcinoma: Chinese College of Interventionalists definition and consensus statement. Chin. Med. J..

[20] Dou Z., Lu F., Ren L., Song X., Li B., Li X. (2022). Efficacy and safety of microwave ablation and radiofrequency ablation in the treatment of hepatocellular carcinoma: A systematic review and meta-analysis. Medicine.

[21] Izzo F., Granata V., Grassi R., Fusco R., Palaia R., Delrio P., Carrafiello G., Azoulay D., Petrillo A., Curley S.A. (2019). Radiofrequency Ablation and Microwave Ablation in Liver Tumors: An Update. Oncologist.

[22] Hwang S., Kim J.H., Yu S.J., Lee J.M. (2024). Incremental high power radiofrequency ablation with multi-electrodes for small hepatocellular carcinoma: A prospective study. BMC Gastroenterol..

[23] Tamai H., Okamura J. (2021). New next-generation microwave thermosphere ablation for small hepatocellular carcinoma. Clin. Mol. Hepatol..

[24] Dong Z., Zhao M., Chen J., Zhao Y., Sun X., Huang B. (2025). Pulsed microwave ablation reduces complications in hepatocellular carcinoma located in high-risk areas: A retrospective comparative study. Int. J. Hyperth..

[25] Han S., Lee J.M., Lee D.H., Yoon J.H., Chang W. (2021). Utility of Real-time CT/MRI-US Automatic Fusion System Based on Vascular Matching in Percutaneous Radiofrequency Ablation for Hepatocellular Carcinomas: A Prospective Study. Cardiovasc. Interv. Radiol..

[26] Odisio B.C., Albuquerque J., Lin Y.M., Anderson B.M., O’Connor C.S., Rigaud B., Briones-Dimayuga M., Jones A.K., Fellman B.M., Huang S.Y. (2025). Software-based versus visual assessment of the minimal ablative margin in patients with liver tumours undergoing percutaneous thermal ablation (COVER-ALL): A randomised phase 2 trial. Lancet Gastroenterol. Hepatol..

[27] Paolucci I., Overduin C.G., Johnston E.W., Laimer G., Ahmed M., Arellano R.S., Beerman M., Beyer L.P., Breen D.J., Burgmans M.C. (2026). International multisociety Delphi consensus for liver tumour thermal ablation: Margin assessment. Lancet Oncol..

[28] Tamai Y., Fujiwara N., Tanaka T., Mizuno S., Nakagawa H. (2023). Combination Therapy of Immune Checkpoint Inhibitors with Locoregional Therapy for Hepatocellular Carcinoma. Cancers.

[29] Sangro B., Kudo M., Erinjeri J.P., Qin S., Ren Z., Chan S.L., Arai Y., Heo J., Mai A., Escobar J. (2025). Durvalumab with or without bevacizumab with transarterial chemoembolisation in hepatocellular carcinoma (EMERALD-1): A multiregional, randomised, double-blind, placebo-controlled, phase 3 study. Lancet.

[30] Kudo M., Ren Z., Guo Y., Han G., Lin H., Zheng J., Ogasawara S., Kim J.H., Zhao H., Li C. (2025). Transarterial chemoembolisation combined with lenvatinib plus pembrolizumab versus dual placebo for unresectable, non-metastatic hepatocellular carcinoma (LEAP-012): A multicentre, randomised, double-blind, phase 3 study. Lancet.

[31] Mariussi M., Gallo Ruelas M., Costa de Oliveira Lima L., Furtado Leite F., Juliano Silva Cunha M., Guedes Moreira Valle L., Boueri Affonso B., Galastri F.L., Serrano Uson Junior P.L., Yuri Itaya Yamaga L. (2025). Selective Internal Radiation Therapy Combined with Immune Checkpoint Inhibitors in the Treatment of Hepatocellular Carcinoma: A Systematic Review and Single-Arm Meta-Analysis. Dig. Dis. Sci..

[32] Yeo Y.H., Liang J., Lauzon M., Luu M., Noureddin M., Ayoub W., Kuo A., Sankar K., Gong J., Hendifar A. (2023). Immunotherapy and Transarterial Radioembolization Combination Treatment for Advanced Hepatocellular Carcinoma. Am. J. Gastroenterol..

[33] Qin S., Chen M., Cheng A.L., Kaseb A.O., Kudo M., Lee H.C., Yopp A.C., Zhou J., Wang L., Wen X. (2023). Atezolizumab plus bevacizumab versus active surveillance in patients with resected or ablated high-risk hepatocellular carcinoma (IMbrave050): A randomised, open-label, multicentre, phase 3 trial. Lancet.

[34] Yopp A., Chen M., Cheng A.L., Kaseb A., Kudo M., Lee H.C., Cha E., Hack S.P., Lian Q., Spahn J. (2026). Updated data from IMbrave050: Adjuvant atezolizumab plus bevacizumab for high-risk hepatocellular carcinoma. J. Hepatol..

[35] Dhondt E., Lambert B., Hermie L., Huyck L., Vanlangenhove P., Geerts A., Verhelst X., Aerts M., Vanlander A., Berrevoet F. (2022). (90)Y Radioembolization versus Drug-eluting Bead Chemoembolization for Unresectable Hepatocellular Carcinoma: Results from the TRACE Phase II Randomized Controlled Trial. Radiology.

[36] Jiang C., Cheng G., Liao M., Huang J. (2021). Individual or combined transcatheter arterial chemoembolization and radiofrequency ablation for hepatocellular carcinoma: A time-to-event meta-analysis. World J. Surg. Oncol..

[37] Wah T.M., Pech M., Thormann M., Serres X., Littler P., Stenberg B., Lenton J., Smith J., Wiggermann P., Planert M. (2023). A Multi-centre, Single Arm, Non-randomized, Prospective European Trial to Evaluate the Safety and Efficacy of the HistoSonics System in the Treatment of Primary and Metastatic Liver Cancers (#HOPE4LIVER). Cardiovasc. Interv. Radiol..

[38] Ziemlewicz T.J., Critchfield J.J., Mendiratta-Lala M., Wiggermann P., Pech M., Serres-Creixams X., Lubner M., Wah T.M., Littler P., Davis C.R. (2025). The #HOPE4LIVER Single-arm Pivotal Trial for Histotripsy of Primary and Metastatic Liver Tumors: One-year Update of Clinical Outcomes. Ann. Surg..

[39] Xu M., Zhang W., Xu D., Dong G., Ren Z., Aji T., Ji J., Zhao Q., Pan J., Chen X. (2025). Nanosecond pulsed electric field ablation as first-line curative therapy for hepatocellular carcinoma in high-risk locations a prospective multicenter. Int. J. Surg..

[40] Vidal-Jove J., Serres X., Vlaisavljevich E., Cannata J., Duryea A., Miller R., Merino X., Velat M., Kam Y., Bolduan R. (2022). First-in-man histotripsy of hepatic tumors: The THERESA trial, a feasibility study. Int. J. Hyperth..

[41] Qu S., Worlikar T., Felsted A.E., Ganguly A., Beems M.V., Hubbard R., Pepple A.L., Kevelin A.A., Garavaglia H., Dib J. (2020). Non-thermal histotripsy tumor ablation promotes abscopal immune responses that enhance cancer immunotherapy. J. Immunother. Cancer.

[42] Yeats E., Lu N., Sukovich J., Xu Z., Hall T.L. (2025). Aberration correction expands the treatment envelope for histotripsy in the liver. Phys. Med. Biol..

[43] Sutter O., Calvo J., N’Kontchou G., Nault J.C., Ourabia R., Nahon P., Ganne-Carrie N., Bourcier V., Zentar N., Bouhafs F. (2017). Safety and Efficacy of Irreversible Electroporation for the Treatment of Hepatocellular Carcinoma Not Amenable to Thermal Ablation Techniques: A Retrospective Single-Center Case Series. Radiology.

[44] Wada T., Sugimoto K., Sakamaki K., Takahashi H., Kakegawa T., Tomita Y., Abe M., Yoshimasu Y., Takeuchi H., Itoi T. (2023). Comparisons of Radiofrequency Ablation, Microwave Ablation, and Irreversible Electroporation by Using Propensity Score Analysis for Early Stage Hepatocellular Carcinoma. Cancers.

[45] Arciga B.M., Walters D.M., Kimchi E.T., Staveley-O’Carroll K.F., Li G., Teixeiro E., Rachagani S., Kaifi J.T. (2025). Pulsed electric field ablation as a candidate to enhance the anti-tumor immune response to immune checkpoint inhibitors. Cancer Lett..

[46] Djokic M., Cemazar M., Bosnjak M., Dezman R., Badovinac D., Miklavcic D., Kos B., Stabuc M., Stabuc B., Jansa R. (2020). A Prospective Phase II Study Evaluating Intraoperative Electrochemotherapy of Hepatocellular Carcinoma. Cancers.

[47] Djokic M., Cemazar M., Popovic P., Kos B., Dezman R., Bosnjak M., Zakelj M.N., Miklavcic D., Potrc S., Stabuc B. (2018). Electrochemotherapy as treatment option for hepatocellular carcinoma, a prospective pilot study. Eur. J. Surg. Oncol..

[48] Kolck J., Schulze D., Bronnimann M., Furstner M., Fehrenbach U., Collettini F., Gebauer B., Auer T.A. (2024). Percutaneous Cryoablation in the Liver: A Meta-Analysis and Review of Safety with a Focus on Incidence of Cryoshock and Major Complications. Cardiovasc. Interv. Radiol..

[49] Hu K.Q. (2014). Advances in clinical application of cryoablation therapy for hepatocellular carcinoma and metastatic liver tumor. J. Clin. Gastroenterol..

[50] Chen Z., Meng L., Zhang J., Zhang X. (2023). Progress in the cryoablation and cryoimmunotherapy for tumor. Front. Immunol..

[51] Gu J., Yu Z., Tang X., Chen W., Deng X., Zhu X. (2024). Cryoablation combined with dual immune checkpoint blockade enhances antitumor efficacy in hepatocellular carcinoma model mice. Int. J. Hyperth..

[52] Sehmbi A.S., Froghi S., Oliveira de Andrade M., Saffari N., Fuller B., Quaglia A., Davidson B. (2021). Systematic review of the role of high intensity focused ultrasound (HIFU) in treating malignant lesions of the hepatobiliary system. HPB.

[53] Wang Y.B., Ma R., Wang Z.B., Shi Q.L., Zhang L., Chen W.Z., Gong J.P., Bai J. (2022). Transcatheter Arterial Chemoembolization in Combination with High-Intensity Focused Ultrasound for Intermediate and Advanced Hepatocellular Carcinoma: A Meta-Analysis. Front. Oncol..

[54] Lyon P.C., Gray M.D., Mannaris C., Folkes L.K., Stratford M., Campo L., Chung D.Y.F., Scott S., Anderson M., Goldin R. (2018). Safety and feasibility of ultrasound-triggered targeted drug delivery of doxorubicin from thermosensitive liposomes in liver tumours (TARDOX): A single-centre, open-label, phase 1 trial. Lancet Oncol..

[55] Abraham R.J., Arepally A., Liu D., Lewandowski R., Kappadath S.C., Verma A., Dobrowski D., Holden A. (2024). Imageable Radioembolization Microspheres for Treatment of Unresectable Hepatocellular Carcinoma: Interim Results from a First-in-Human Trial. J. Vasc. Interv. Radiol..

[56] Roosen J., Westlund Gotby L.E.L., Arntz M.J., Futterer J.J., Janssen M.J.R., Konijnenberg M.W., van Wijk M.W.M., Overduin C.G., Nijsen J.F.W. (2022). Intraprocedural MRI-based dosimetry during transarterial radioembolization of liver tumours with holmium-166 microspheres (EMERITUS-1): A phase I trial towards adaptive, image-controlled treatment delivery. Eur. J. Nucl. Med. Mol. Imaging.

[57] Solymos P., Redei M., Turan C., Szabo B., Adam A., Molnar Z., Duray G., Hegyi P., Horvathy D.B. (2025). Holmium-166 Radioembolization Is a Safe and Effective Locoregional Treatment for Primary and Secondary Liver Tumors: A Systematic Review and Meta-Analysis. Cancers.

[58] Stella M., Braat A., van Rooij R., de Jong H., Lam M. (2022). Holmium-166 Radioembolization: Current Status and Future Prospective. Cardiovasc. Interv. Radiol..

[59] Jaroch D.B., Liu Y., Kim A.Y., Katz S.C., Cox B.F., Hullinger T.G. (2025). Pressure-Enabled Drug Delivery Significantly Increases Intra-Arterial Delivery of Embolic Microspheres to Liver Tumors in a Porcine Model. J. Vasc. Interv. Radiol..

[60] Goh G.S., Goodwin M.D., Huang J.F., Kavnoudias H., Holden A. (2022). A Pilot First-in-Human Study of Embrace, a Polyethylene Glycol-Based Liquid Embolic Agent, in the Embolization of Malignant and Benign Hypervascular Tumors. J. Vasc. Interv. Radiol..

[61] Jia W., Han Y., Mao X., Xu W., Zhang Y. (2022). Nanotechnology strategies for hepatocellular carcinoma diagnosis and treatment. RSC Adv..

[62] Kim H., Choi B., Mouli S.K., Choi H., Harris K.R., Kulik L.M., Lewandowski R.J., Kim D.H. (2023). Preclinical Development and Validation of Translational Temperature Sensitive Iodized Oil Emulsion Mediated Transcatheter Arterial Chemo-Immuno-Embolization for the Treatment of Hepatocellular Carcinoma. Adv. Healthc. Mater..

[63] Negussie A.H., Mikhail A.S., Owen J.W., Hong N., Carlson C.J., Tang Y., Carrow K.P., Mauda-Havakuk M., Lewis A.L., Karanian J.W. (2022). In vitro characterization of immune modulating drug-eluting immunobeads towards transarterial embolization in cancer. Sci. Rep..

[64] de Baere T., Roux C., Deschamps F., Tselikas L., Guiu B. (2022). Evaluation of a New CT-Guided Robotic System for Percutaneous Needle Insertion for Thermal Ablation of Liver Tumors: A Prospective Pilot Study. Cardiovasc. Interv. Radiol..

[65] Heerink W.J., Ruiter S.J.S., Pennings J.P., Lansdorp B., Vliegenthart R., Oudkerk M., de Jong K.P. (2019). Robotic versus Freehand Needle Positioning in CT-guided Ablation of Liver Tumors: A Randomized Controlled Trial. Radiology.

[66] Tinguely P., Paolucci I., Ruiter S.J.S., Weber S., de Jong K.P., Candinas D., Freedman J., Engstrand J. (2021). Stereotactic and Robotic Minimally Invasive Thermal Ablation of Malignant Liver Tumors: A Systematic Review and Meta-Analysis. Front. Oncol..

[67] Evans M., Kang S., Bajaber A., Gordon K., Martin C. (2025). Augmented Reality for Surgical Navigation: A Review of Advanced Needle Guidance Systems for Percutaneous Tumor Ablation. Radiol. Imaging Cancer.

[68] Kiani I., Razeghian I., Valizadeh P., Esmaeilian Y., Jannatdoust P., Khosravi B. (2026). Performance of Artificial Intelligence Models in Predicting Responsiveness of Hepatocellular Carcinoma to Transarterial Chemoembolization (TACE): A Systematic Review and Meta-Analysis. J. Am. Coll. Radiol..

[69] Tarantino L., Busto G., Nasto A., Fristachi R., Cacace L., Talamo M., Accardo C., Bortone S., Gallo P., Tarantino P. (2017). Percutaneous electrochemotherapy in the treatment of portal vein tumor thrombosis at hepatic hilum in patients with hepatocellular carcinoma in cirrhosis: A feasibility study. World J. Gastroenterol..

